# Editorial: Digital suicide prevention

**DOI:** 10.3389/fdgth.2023.1148356

**Published:** 2023-03-03

**Authors:** Lasse Bosse Sander, Lena Spangenberg, Louise La Sala, Wouter Van Ballegooijen

**Affiliations:** ^1^ Medical Psychology and Medical Sociology, Faculty of Medicine, University of Freiburg, Freiburg, Germany; ^2^ Department of Medical Psychology and Medical Sociology, Leipzig University, Leipzig, Germany; ^3^ Orygen, Centre for Youth Mental Health, University of Melbourne, Parkville, VIC, Australia; ^4^ Department of Clinical, Neuro and Developmental Psychology & Department of Psychiatry, Amsterdam UMC, Vrije Universiteit Amsterdam, Amsterdam, Netherlands

**Keywords:** suicide, ecological momentary assessment (EMA), prevention, prediction, technology

**Editorial on the Research Topic** Digital Suicide Prevention

Suicide is a major public health concern and a leading cause of death in young people around the world, with a global mortality rate of over 700,000 people per year (1). Despite advances in mental health care and suicide prevention efforts, suicide rates continue to be high (1). In addition, the ability to predict suicides has not progressed in the last five decades of research, also because studies repeatedly investigated the same set of potential risk factors in most trials and used assessments, which do not adequately take the complexity of suicidality into account (2). Suicidal ideation is a complex condition that can manifest both within and outside of various mental health and physical health disorders (3–6). Furthermore, the fluctuation of suicidal ideation and associated risk factors within hours or days necessitate more precise and detailed assessments (7, 8). Thus, to make progress in suicide research, multiple underlying processes or mechanisms may need to be investigated.

The digital era has brought a range of opportunities and methodological advances for comprehending, predicting, and averting suicidal behaviour. Ecological Momentary Assessment (EMA) allows for the timely identification of suicidal ideation and potentially related risk factors in a naturalistic setting (9). The utilization of machine learning techniques presents a unique approach for the analysis of intricate datasets (10). Furthermore, digital technologies can provide access to support and information, allow for early identification and intervention, and facilitate data-driven approaches to suicide prevention (11–15).

This Research Topic, *Digital Suicide Prevention*, has brought together seven articles that explore the potential of digital technologies to reduce suicide-related harm from a range of international experts.

In one study, Cohen et al. evaluate the performance of a machine learning suicide risk prediction model in the emergency department setting. The results show that the language-based suicide risk model performed with good discrimination in an external validation set.

A second study (Janssen et al.) explores whether motivational interviewing is an improvement over the five-phase model for a chat-based suicide-prevention helpline. The authors conclude that motivational interviewing adds more structure but does not seem to be associated with greater symptom reductions. Several next steps to improve in this field are described.

Three articles deal with different aspects of the use of EMA in suicide research. Based on the widespread use of smartphones and immense technological advancements, the number and quality of EMA studies has greatly increased during the last decade. Kivelä et al. review the current evidence on EMA in suicide research highlighting main findings (i.e., fluctuation of suicide ideation, general feasibility of EMA) and open questions (i.e., balancing study design and ethical/ safety issues). Ernst et al. present a study protocol for a study investigating the transdiagnostic risk and resilience factors associated with suicidal ideation using EMA. Brüdern et al. introduce the dual-system model of suicidality, which takes new directions to conceptualize suicidality and advertise EMAs as a well-suited methodological approach to investigate the proposed processes in detail.

In another study, Benson et al. present a dashboard prototype for real-time suicide mortality data using a dataset from the Suicide Support and Information System in Southern Ireland. This intuitive interface integrates a cluster detection approach and demonstrates real-world applicability as a proactive monitoring tool for timely action in suicide prevention.

Finally, Hopkins et al. review the use of machine learning prediction models in structured (human interpretable such as psychometric instruments) vs. unstructured (only machine interpretable such as electronic health records) datasets. The meta-analysis indicates that structured data and unstructured data showed similar outcome accuracy, despite different levels of data organization and specificity regarding the outcome of suicide risk prediction.

The articles in this Research Topic provide valuable insights into the potential of digital technologies to aid in understanding suicide and suicide prevention initiatives. The content of the articles reflects a diverse range of digital suicide prevention techniques, including but not limited to EMA, crisis chat services, and machine learning algorithms. Yet, it shows that each of these facets is only just developing and has a long way ahead to reach its full potential. Digital technologies can be quickly adapted to changing contexts and have the potential to reach large numbers of people. Online or app-based support might even reach those who are reluctant to seek help or those who cannot easily find mental healthcare. However, these technologies must be developed in an ethical and responsible way, with due consideration of the potential harms that may arise from the use of digital technologies (16). All authors critically discuss current boundaries and potential barriers related to the use of digital technologies and point out the next challenges for the field. We would like to thank the authors for their contributions to this Research Topic, and we look forward to engaging in further dialogue and collaboration on this important topic.
